# Canine Mammary Tumors: Classification, Biomarkers, Traditional and Personalized Therapies

**DOI:** 10.3390/ijms25052891

**Published:** 2024-03-01

**Authors:** Natalia Nosalova, Mykhailo Huniadi, Ľubica Horňáková, Alexandra Valenčáková, Slavomir Horňák, Kamil Nagoos, Juraj Vozar, Dasa Cizkova

**Affiliations:** Small Animal Clinic, University of Veterinary Medicine and Pharmacy, Komenskeho 73, 041 81 Kosice, Slovakia; natalia.nosalova@uvlf.sk (N.N.); mykhailo.huniadi@uvlf.sk (M.H.); lubica.hornakova@uvlf.sk (Ľ.H.); avalencakova@uvlf.sk (A.V.); slavomir.hornak@uvlf.sk (S.H.); kamil.nagoos@student.uvlf.sk (K.N.); juraj.vozar@student.uvlf.sk (J.V.)

**Keywords:** canine mammary tumor, biomarkers, treatment, personalized medicine

## Abstract

In recent years, many studies have focused their attention on the dog as a proper animal model for human cancer. In dogs, mammary tumors develop spontaneously, involving a complex interplay between tumor cells and the immune system and revealing several molecular and clinical similarities to human breast cancer. In this review, we summarized the major features of canine mammary tumor, risk factors, and the most important biomarkers used for diagnosis and treatment. Traditional therapy of mammary tumors in dogs includes surgery, which is the first choice, followed by chemotherapy, radiotherapy, or hormonal therapy. However, these therapeutic strategies may not always be sufficient on their own; advancements in understanding cancer mechanisms and the development of innovative treatments offer hope for improved outcomes for oncologic patients. There is still a growing interest in the use of personalized medicine, which should play an irreplaceable role in the research not only in human cancer therapy, but also in veterinary oncology. Moreover, immunotherapy may represent a novel and promising therapeutic option in canine mammary cancers. The study of novel therapeutic approaches is essential for future research in both human and veterinary oncology.

## 1. Canine Mammary Tumors

### 1.1. General Information and Incidence 

Canine mammary tumors (CMTs) represent a serious clinical problem in veterinary medicine because they are among the most frequent carcinomas that occur in female dogs [1]. Among the many studies, Schneider (1970) described the annual incidence rate for CMTs of 145 cases per 100,000 female dogs in California, USA [2]. In 2005–2013, analysis of CMTs in the Venice and Vicenza provinces showed a standardized incidence rate of 250/100,000 dogs/year. Recently, Varney et al. (2023) reported the frequency of CMTs in bitches as 1340.7 per 100,000 dogs in 2016 in the UK [3]. A study by Zheng et al. (2022) showed that the relative incidence of CMTs in Mainland China between 2017–2021 was 46.71%, with a similar number of malignant and benign forms [4]. In the female dog population living in northeastern Italy, CMTs made up 54% of all tumors in bitches [5]. Several studies have shown that the incidence of CMTs differs between regions [6]. Recently, CMT frequency has decreased in developed countries due to reproductive health programs [7]. For example, in regions such as Western Europe or the United States, the incidence of CMTs is diminishing, because ovariohysterectomy is routinely performed at an early age there. On the other hand, Spain and Scandinavia show a higher incidence of CMTs. In these regions, preventive neutering is not a common procedure in dogs [6]. Currently, it is known that ovariohysterectomy significantly reduces the risk of cancer development [8]. 

### 1.2. Classification and Immunohistochemical Markers 

In general, cancers can be divided into benign and malignant forms. Although it is difficult to distinguish benign and malignant mammary tumors, there are some differences between them. Typical features of malignant mammary tumors are an irregular chromatin pattern, destructive growth, discontinuous basal membranes, invasion of vessels, necrosis, high mitotic index, and cellular polymorphism. On the other hand, benign mammary tumors are often encapsulated and lack invasive growth [6,9]. The World Health Organization (WHO) approved the official histological classification for CMTs, which was used for many years worldwide [10]. In 2011, Goldschmidt and co-workers created a new complete classification scheme, according to which CMTs were divided into 23 malignant and 7 benign subtypes [11]. This classification system was subsequently updated in 2019 by WHO and the Davis-Thompson DVM Foundation (Table 1) [12,13]. Malignant CMTs are mostly adenocarcinoma, carcinosarcoma, solid carcinoma, papillary carcinoma, or complex carcinoma. The most common benign forms of CMTs are ductal papillomas, fibroadenomas, simple adenomas, and benign mixed tumors [7,14]. Approximately more than 50% of CMTs are histologically malignant [15].

The origin of CMTs is mostly from epithelial tissues (simple carcinoma/simple adenoma), though a few tumors are a combination of epithelial and myoepithelial origin (complex carcinoma/complex adenoma). Others are of mesenchymal origin (osteosarcoma, fibrosarcoma, fibroadenoma), and carcinosarcoma or mixed benign tumors are from both mesenchymal and epithelial tissues [6,9,10].

Mammary cancers are also classified into four subtypes according to the expression of immunohistochemical markers including estrogen receptor (ER), progesterone receptor (PR), and receptor of human epidermal growth factor 2 (HER-2 or EGFR-2): I. Luminal A (ER^+^ and/or PR^+^, and HER-2^−^), II. Luminal B (ER^+^ and/or PR^+^, and HER-2^+^), III. HER-2 positive (ER^−^, PR^−^ and HER-2^+^), and IV. Triple-negative (ER^−^, PR^−^ and HER-2^−^) [16,17]. In humans, HER-2 is detected based on gene amplification with the method of fluorescence in situ hybridization (FISH) and protein expression using immunohistochemistry. The assessment of HER-2 status in human breast cancer is revised by experts from the American Society of Clinical Oncology and the College of American Pathologists (ASCO/CAP) [18]. In veterinary medicine, the biological meaning of HER-2 overexpression in CMTs is not straightforward. The amplification status of HER-2 was investigated previously in CMT via chromogenic in situ hybridization; however, no HER-2 amplification was detected [19]. Muscatello and co-workers performed single- and dual-probe fluorescent in situ hybridization and evaluated chromosomal abnormalities, translocations, co-amplification, or polysomy. In their study, a significant correlation was demonstrated between HER-2 overexpression and increased numbers of HER2 gene copies, but there was no correlation with the amplification status determined by the ASCO/CAP evaluation system [20]. This indicated that the evaluation system for human breast cancer is not adaptable for CMT, and future studies are needed to clarify the clinical role of HER-2 in canines.

Several immunohistochemical markers of CMTs have already been investigated to provide a better understanding of tumor biology. Aside from ER, PR, and HER-2, the panels of these markers also include cytokeratins (CKs), β-tubulins, mucin-1, Ki-67, p53, calponin, vimentin, smooth muscle actin (SMA), and p63 [17,21,22,23]. Some markers will be described in more detail in this section, and a few of them will be mentioned below in the subsection Biomarkers. 

Actin microfilaments, intermediate filaments, and microtubules are involved in the formation of the cytoskeleton in mammalian cells. Cytokeratins (CKs) belong to the intermediate filaments whose expression reflects cell differentiation, tissue growth status, and epithelial cell type [24]. In normal human and canine mammary glands, the luminal epithelial cells exhibit the expression of CK7, CK8, CK18, and CK19. On the other hand, CK5, CK14, CK15, and CK17 are expressed in the basal/myoepithelial cells [25,26]. In malignant CMT, downregulation of luminal CK19 was associated with an aggressive phenotype and a higher risk of tumor progression [27]. Fhaikrue et al. (2020) demonstrated the expression of CK5 in complex carcinoma and benign mixed tumor cells. Moreover, CK18 was found in simple carcinoma cells [28]. A high expression of CK18 was also identified in solid carcinoma [29]. Immunohistochemical expression of CKs may represent a promising approach in the histological examination of mammary cancer [30].

**Figure 1:**
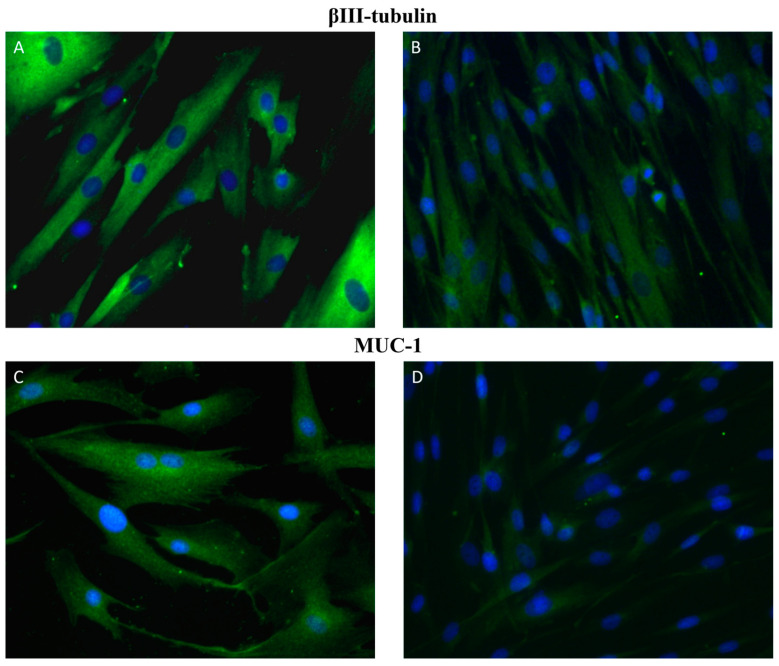
Immunofluorescence staining of primary cultures of canine mammary glands. Representative images of βIII-tubulin (**A**,**B**) and MUC-1 (**C**,**D**) positive cells (green). Images (**A**,**C**) represent primary cultures isolated from CMT that was histologically diagnosed as tubulopapillary carcinoma of mammary gland. Images (**B**,**C**) show healthy non-cancerous cells isolated from canine mammary glands. Magnification: 40×.

Microtubules, critical components of the cell cytoskeleton, are heterodimers of α- and β-tubulins expressed in a tissue-specific manner [31]. βIII-tubulin in particular occurs in neural tissue and regulates neuronal microtubule function [32]. Although βIII-tubulin is not present in normal mammary epithelium, it is highly expressed in breast, ovarian, colon, and lung cancers. Furthermore, overexpression of βIII-tubulin was associated with poor prognosis of malignancies [33,34,35,36]. Kanojia et al. (2015) demonstrated the implication of βIII-tubulin in the development of brain metastasis in patients with breast cancer [37]. Another isotype of microtubules is class II β-tubulin, which was expressed in myoepithelial cell lines created from canine mammary mixed tumors [23]. Moreover, immunofluorescence analysis of primary cultures isolated from CMT that was histologically diagnosed as tubulopapillary carcinoma showed higher positivity of cells to βIII-tubulin than healthy non-cancerous cells isolated from the canine mammary gland (Figure 1). Although there are no studies regarding βIII-tubulin as a marker of CMTs, dogs are considered a suitable animal model for breast cancer; therefore, we believe that βIII-tubulin could be a promising target in the diagnosis of canine mammary cancer. 

Circulating biomarkers, including glycoprotein substances, can be detected in blood under both normal healthy and neoplastic conditions. However, their levels are significantly increased in carcinomas, thus they are considered as tumor indicators [38]. Mucin-1 (MUC-1), also known as cancer antigen 15-3 (CA15-3) is a heavily glycosylated protein with high molecular weight produced by epithelial cells. The physiological functions of MUC-1 include epithelial cell protection, cellular adhesion, lubrication, and others [39]. Under normal conditions, MUC-1 is localized on the apical surface, but loss of apical localization occurs during malignant transformation [40]. Over-expression of MUC-1 was found in many cancers and was related to increased tumor invasiveness and poor prognosis [39,40]. Gene mutations, such as two deletions (in intron 6 and exon 7) and three single-nucleotide polymorphisms, were found in canine MUC-1 [41]. In CMTs, MUC-1 expression was also associated with tumor invasion and the presence of metastases, which had prognostic significance [22,42,43]. The study of Manuali et al. (2012) demonstrated that high expression of MUC-1 was positively correlated with tumor grade [44]. In several studies, a significant increase in the serum concentration of MUC-1 was found in dogs with CMTs compared to dogs with no evidence of mammary neoplasia [42,45]. Similarly, immunocytochemistry showed increased expression of MUC-1 in primary cultures of CMT cells originating from tubulopapillary carcinoma of the mammary gland compared with healthy non-cancerous mammary gland cells (Figure 1). Moreover, higher serum levels of MUC-1 in canine were related to regional and distant metastases [46]. 

Mucins can also be involved in cellular and humoral immunity. They can block natural killer (NK) cells, cytotoxic T-lymphocyte (CTL) cells, and neutrophil activity, which results in the inhibition of anticancer immune response. MUC-1 suppresses the ability of immune cells to bind to their target and consequently prevents immunotherapy effectiveness in tumors [47]. Some techniques, such as increasing the efficacy of vaccines, have been used to enhance immune response to MUC-1 [48]. We will mention this in more detail in the section on personalized medicine. MUC-1 is one of the most widely used serum tumor biomarkers in women with breast cancer and recent studies suggest its promising potential in the diagnosis and treatment of CMTs [44]. 

### 1.3. Grading System and Clinical Staging

In veterinary medicine, different grading systems are used. In general, malignant CMTs were classified by Misdorp (2002) [9], who adopted the human grading system of Bloom and Richardson (1975) [49]. For several years, the most applied grading method for CMTs, described by Elston and Ellis (1991), has been the Nottingham histological grade [50]. This system was modified according to the Bloom and Richardson method. Both grading systems include three parameters: mitotic counts, nuclear pleomorphism, and tubule formation. Each parameter is evaluated in a range from 1 to 3, and the histological malignancy grade is determined by the sum of particular scores [51]. It is known that three grades of malignancy exist for CMTs: I. grade (well-differentiated)—total score is from 3 to 5; II. Grade (moderately differentiated)—total score 6–7; and III. Grade (poorly differentiated)—total score is 8–9 (Table 2) [9,50]. Recently, Peña and co-workers presented another grading method for CMTs, where they included all types of cells for the evaluation of the histological grade. This system involved modifications in the appointment of two parameters—mitotic count and nuclear pleomorphism [52]. Grading systems are important for the prognosis of CMTs, and a higher grade of malignancy means a worse prognosis for patients [6].

The clinical staging of CMTs is determined according to the TNM system approved by the WHO, which defines the extension of CMT based on the primary tumor size (T), involvement of lymph nodes (N), and the presence of distant metastasis (M). One of the most important prognostic factors for CMTs is tumor size categorized into three groups: T1—smaller than 3 cm, T2—3–5 cm, and T3—greater than 5 cm. Larger tumors (T3) were associated with a higher proliferation rate and risk of malignancy [53,54,55]. The status of regional lymph nodes is classified based on the presence or absence of metastasis as N0—no metastasis and N1—presence metastasis [21]. Assessment of regional lymph nodes is also important for CMT prognosis [55,56,57]. The third essential prognostic factor for CMTs is the detection of distant metastasis (presence—M0, absence—M1), which is most often localized in the lungs of dogs [21].

### 1.4. Risk Factors 

**Figure 2:**
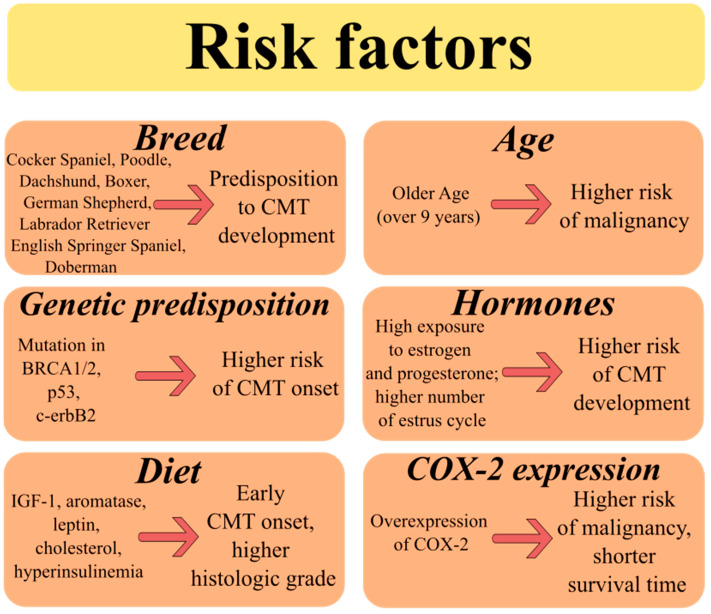
Risk factors of canine mammary cancer. The original figure was created using Inkscape v1.1.2 software.

The most important factors influencing the incidence and development of CMTs are breed, age, genetic predisposition, hormones, diet, and cyclooxygenase-2 expression (Figure 2) [6]. It is known that CMT can occur in any breed of dog, but more cases were found in pure breeds [11,21]. Cocker Spaniel, Poodle, Dachshund, German Shepherd, and Labrador Retriever are among the most affected bitches, and they have a predisposition to tumor development [7,58]. A study from Sweden reported that 36% of English Springer Spaniels had a high risk of CMT onset [59]. Similarly, Egenvall (2005) showed that English Springer Spaniels, Boxers, and Dobermans were predisposed to mammary cancer development [60].

CMT usually occurs in middle-aged and elderly dogs between 8–10 years old [21]. Recent studies have shown some differences in the development of malignant and benign tumors. While the benign forms frequently affect dogs at the age of 7–9 years old, malignant forms are more likely to develop in older bitches [5,61,62]. Burrai and co-workers (2020) also described that female dogs with benign CMT were significantly younger than those with malignant tumors (8.7 vs. 9.6 years) [63].

In women, a high risk of developing breast cancer is represented by mutations in the BRCA1 and BRCA2 genes. In veterinary medicine some discrepancies exist between studies that consider mutation in these genes as a risk factor for CMTs. Rivera et al. (2009) detected 10 different genes in dogs with CMT, but only BRCA1 and BRCA2 were significantly involved in cancer progression [59]. The study by Nieto and co-workers (2003) found an association between BRCA1 mutation and the malignant form of CMT [57]. On the other hand, a decrease in BRCA2 gene expression was also described [64]. Since knowledge about gene expression of BRCA1/2 in CMTs is still unclear, further studies need to be carried out on the genome and mutations in canine [15]. In addition, oncogenes *p53* and *c-erbB2* have also been evaluated in CMTs. Several studies described the overexpression of mutant *p53* and *c-erbB2* as being involved in carcinogenesis in dogs [65,66,67].

Mammary tumors are controlled by ovarian hormones, both estrogens, and progesterone, and increased exposure to them, especially during the estrus cycle, may be involved in the development of CMTs [6]. These hormones elevate the proliferation of canine mammary cancer cells through hormone steroid receptor activation [68]. Bitches neutered before their first estrus have the lowest risk of CMTs onset (0.5%) [69]. This is probably related to a reduction in the occurrence of proliferation stimuli and therefore the risk for mutations. Each additional estrus cycle increases the risk of developing CMTs [63]. Growth factors, such as insulin-like growth factor 1 (IGF-1) and adipocytes, which release aromatase, leptin, and cholesterol, can also be sources of sex hormones [70]. Insulin resistance, hyperinsulinemia, and increased levels of IGF-1 are often associated with obesity and can lead to rapid growth and invasion of breast cancers [71]. The anti-apoptotic and mitogenic effects of IGF-1 on CMTs are mediated by binding with its receptor IGFR-1 as well as increasing the estrogen receptor activation [72]. Leptin stimulates the proliferation of cancer cells through upregulation of aromatase gene expression [73]. In the study of Marinelli et al. (2004) aromatase expression was found to be significantly higher in CMT compared with normal mammary tissue [74]. Overweight female dogs had an increased expression of aromatase, early CMT onset, and a higher histologic grade. Taken together, obesity, an accumulation of adipocytes, represents an important risk factor for CMT development [70].

Prostaglandins are synthesized from arachidonic acid by the enzyme cyclooxygenase, which can exist as two isoforms (COX-1 and COX-2) with distinct biological functions. In general, COX-1, the constitutive form present in many tissues, regulates normal physiological functions, whereas COX-2 refers to an inducible form that is stimulated by inflammatory responses, growth factors, or oncogenes [75]. Isoenzyme COX-2 may be involved in carcinogenesis through the promotion of angiogenesis, invasion, metastasis, or suppression of apoptosis [76]. Overexpression of COX-2 has been found in many types of cancers in humans as well as in animals [77,78,79,80]. Different studies showed that elevated levels of COX-2 in CMTs may be one of the important prognostic factors [81,82,83]. An increased COX-2 expression in dogs with CMTs was related to shorter survival time and worse prognosis [84]. Doré and co-workers (2003) demonstrated for the first time that the expression of COX-2 is higher and more frequent in malignant CMTs compared with benign ones [85]. These differences between malignant and benign forms were confirmed by other studies [86,87,88].

### 1.5. Biomarkers

Biomarkers are biological molecules measured in body fluids (blood, urine) or tissues that can provide some information about disease. Detection of biomarkers is usually used to determine the diagnosis, prognosis, or staging of cancers in both human and veterinary oncology [89]. Ki-67, PCNA, p53, VEGF, EGFR, E-cadherin, COX-2, BRCA-1/2, miRNA, and cancer stem cells (CSCs) are among the most frequently detected biomarkers in CMTs [15], and some of them will be described in this review.

**Figure 3:**
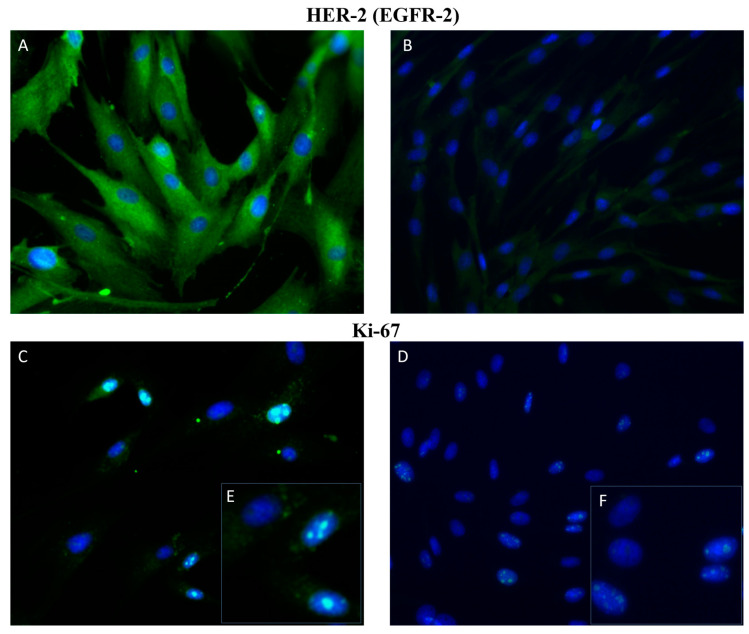
Immunofluorescence staining of primary cultures of canine mammary glands. Representative figures of HER-2 (**A**,**B**) and Ki67 (**C**,**D**) positive cells (green). Images (**A**,**C**) are primary cultures isolated from CMT (tubulopapillary histotype) and (**B**,**D**) primary cultures isolated from healthy non-cancerous canine mammary glands. (**E**,**F**) are a detail of (**C**,**D**). Magnification: 40×.

One of the most studied prognostic biomarkers of CMTs is a non-histone nuclear protein Ki-67. It can be detected in the nucleus of cells during interphase or mitosis, while its expression increases in proliferative cells [90,91]. Ki67 expression is influenced by several factors, such as inflammation of the mammary gland, tumor size, invasion into other tissues, or lymph node metastasis [92,93]. Different studies reported that a high Ki67 expression positively correlated with tumor grade, metastasis, and poor prognosis in dogs. Moreover, significantly lower levels of Ki67 were determined in benign mammary tumors compared with malignant ones [93,94,95]. Furthermore, analysis of primary cultures isolated from CMT using immunofluorescence staining showed Ki67 positive nuclei (Figure 3). Proliferating cell nuclear antigen (PCNA) is also a key marker of cancer cell proliferation. PCNA, DNA polymerase δ auxiliary protein, is expressed in the cell’s nuclei during the S-phase of the cell cycle [96]. The main function of this protein involved not only DNA replication but also the DNA repair process, chromatin remodeling, and cell cycle control [97]. Similar to Ki67, a greater expression of PCNA was found in cancers with large tumor size, higher histological grade, skin ulceration, and the presence of metastasis [98]. Aydogan and co-workers (2018) showed that PCNA expression is a prognostic indicator for canine mammary carcinosarcomas [99].

Protein p53 is known as a biomarker of cell division, apoptosis, and neoplastic transformation. As we mentioned above, mutations of p53 are one of the most frequently occurring genetic alterations in CMTs, and these mutations are involved in tumor progression [100]. In human and veterinary medicine, a high gene expression of *p53* was associated with poor prognosis and shorter overall survival [67,101,102].

A typical feature of neoplastic cell transformation is angiogenesis, which is essential for maintaining homeostasis and supplying the tumor with nutrients [103]. Vascular endothelial growth factor (VEGF) and epidermal growth factor receptors (EGFR1,2) are key biomarkers of angiogenesis. Both VEGF and EGFR are responsible for blood vessel formation, endothelial proliferation, and cell migration [104,105]. Many studies have found a positive correlation between overexpression of VEGF and metastasis formation [106,107,108]. Moreover, VEGF expression is closely correlated with clinical stage and lymph node metastasis in CMTs [109]. An association between high expression of EGFR and tumor malignancy, mitotic index, tumor size, dog’s age, and poor prognosis were also found [110,111]. An increased expression of EGFR-2 (HER-2) was also determined in primary cultures of CMT cells isolated from tubulopapillary carcinoma of the mammary gland compared with primary culture cells isolated from healthy non-cancerous canine mammary glands (Figure 3).

Another valuable group of biomarkers are proteins indicating the tumor metastatic potential, which depends on the ability of cells to adhere to other cells or tissues. Selectins, integrins, cadherins, and immunoglobulin-like particles belong to these adhesion molecules [15]. Cadherins are membrane-bound Ca2+-dependent receptors mediating the cell-cell contacts. E-cadherin (called epithelial cadherin) is one of the most frequently detected cadherin in many cancers [112,113,114]. Several studies described that downregulation of E-cadherin gene expression in CMTs was associated with larger tumor size, high histological grade, invasive growth, and metastasis formation [115,116]. In addition, a significant association between decreased expression of E-cadherin and shorter overall survival was reported [117].

MicroRNAs (miRNAs) are known as small (20–23 nucleotides) non-coding molecules which can modulate gene expressions [118]. First, the primary transcript (pri-miRNA) is synthesized by RNA polymerase II in the nucleus and subsequently cleaved to an approximately 70 nucleotides long precursor miRNA (pre-miRNA). Then, the pre-miRNA is exported to the cytoplasm and used for miRNA duplexes formation. One strand is selected as mature miRNA and the second one is degraded [119]. MiRNAs can regulate the expression of many genes post-transcriptionally and play a crucial role in cellular processes including differentiation, proliferation, migration, angiogenesis, or apoptosis [120,121]. Deregulation of miRNA expression was found in various cancer diseases [122,123,124]. In cancers, miRNAs can be divided into two groups; the first one acts as tumor suppressors, and the second group as oncogenes. Alteration of both tumor suppressor miRNAs, such as *mi-R206*, *miR-17-5p*, *miR-125*, *miR-34a*, *miR-200* or *miR-31*, as well as overexpression of oncogenes miRNA (*miR-21*, *miR-155*, *miR-10b*, *miR-373/520c*), were investigated in human breast cancer [119]. In veterinary medicine, many studies on miRNA as potential biomarkers for CMTs have been reported [125,126,127]. It is generally known that dogs present a model for human breast cancer because of their physiologic and genetic similarity to humans. Furthermore, almost the same miRNA expression pattern was found in CMT compared to human breast cancer [128]. Heishima and co-workers (2017) demonstrated that *miR-126* and *miR-214* were significantly elevated in dogs with CMT, which could have diagnostic and prognostic potential [125]. Another study showed a significant role of *miRNA-143* and *miRNA-138a* in the malignancy of canine mammary cancer cell line [126]. Moreover, overexpression of *miR-18a* and *miR-18b* increased the proliferation of cancer cells in malignant CMTs [127]. MiRNA expression profile differs significantly between non-metastatic and metastatic CMTs [129]. In vitro analysis detected that canine mammary cancer cells may secrete exosomes containing miRNA. These exosomal miRNAs, after being released into the blood, can regulate hormone receptors and some oncogenic pathways [130]. 

MiRNAs functioning in the regulation of differentiation and self-renewal properties of cancer stem cells (CSCs) have also been described [131,132]. CSCs, also known as tumor-initiating cells (TICs), are a small group of cancerous populations important for tumor growth that are capable of renewing themselves and differentiating into various kinds of cancer cells. CSCs also seem to be responsible for resistance to chemotherapy and radiotherapy. Many of these stem cells can induce relapse and formation of distant metastases [133]. CSCs can arise from normal stem cells through neoplastic transformation, mature cancer cells by dedifferentiation, or from pluripotent cancer cell induction [134]. The *let-7* miRNA family plays a crucial role in the phenotype of CSCs. Cai and colleagues (2013) described a downregulation of *let-7* miRNA expression in breast cancer, which led to the expansion of the breast CSCs population [131]. Significant deregulation of the miRNA expression profile was found in canine CSCs compared to differentiated cancer cells [135]. CSCs express specific cell surface antigens (CD24, CD44, EPCAM), which allow their easier identification. Association between the CD44+/CD24− phenotype of CSCs and higher histological grade of CMTs was determined [136]. It is very important to evaluate biomarkers for diagnosis, prognosis, or treatment of cancer disease and miRNAs have promising potential due to their high sensitivity and specificity.

## 2. Treatment of Canine Mammary Tumors

**Figure 4:**
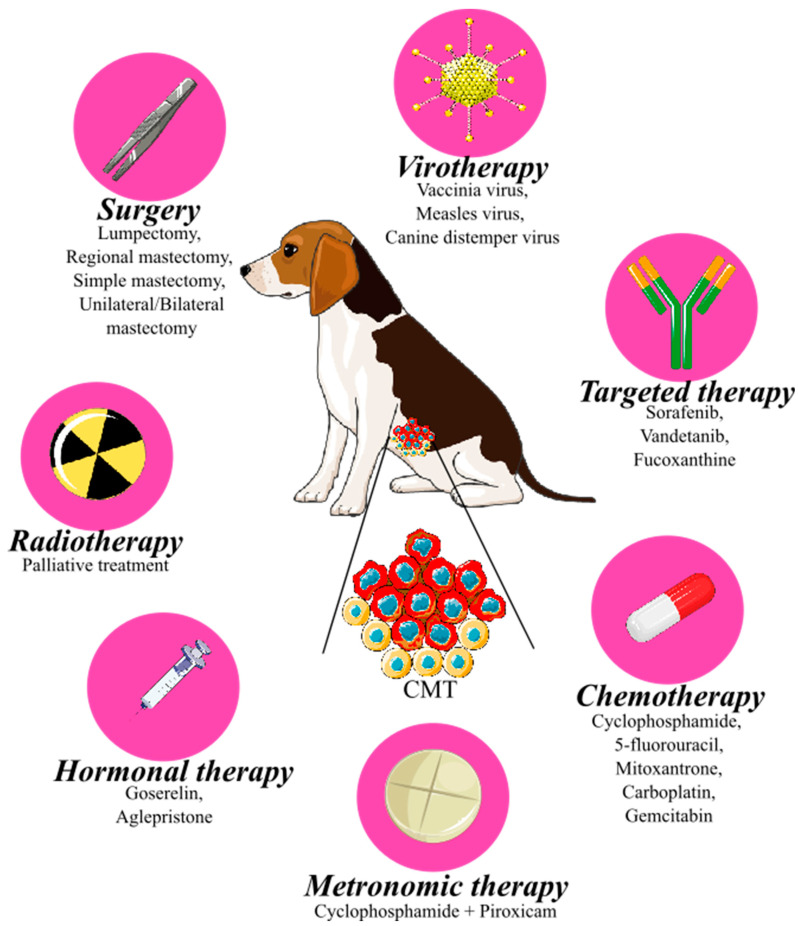
Therapeutic strategy of canine mammary tumor, CMT (red cells = tumor, yellow cells = normal (luminal, basal, stromal) cells of mammary gland). An original figure. Inkscape v1.1.2 software was used to create the figure.

The treatment of CMTs includes surgery, radiotherapy, hormonal therapy, chemotherapy, targeted therapy, and virotherapy (Figure 4). Surgery remains the first choice of treatment for many types of CMTs. Surgical excision is the most effective modality for regional tumor control and includes histological evaluation, which increases overall survival. Lumpectomy, regional mastectomy, simple mastectomy, and unilateral and bilateral mastectomy are among the surgical techniques used [6]. The type of surgery is selected based on certain criteria, including tumor size and location, possible extension to regional lymph nodes, adherence and fixation of tumor to tissue, and total number of lesions [137]. Lumpectomy, or nodulectomy, is indicated to remove small (<0.5–1 cm), firm, and nonfixed benign nodules. During lumpectomy, the skin is incised over the nodule, which is bluntly dissected from the surrounding parenchyma [137]. Simple (single) mastectomy implies the removal of one mammary gland and is indicated for large lesions having a central location in the gland and exhibiting fixation to the underlying tissues or overlying skin [55]. Regional mastectomy is the removal of large mammary tumors with localization in consecutive glands or between two glands. This surgical method was originally proposed based on the concept of venous and lymphatic drainage [55]. The removal of mammary glands 1 to 5 as a unit is performed by unilateral mastectomy, which is indicated for multiple tumors in glands of one chain [137]. Bilateral mastectomy is performed in multiple tumors with localization in both chains [138]. Dogs with lower clinical stages of the disease, small, non-invasive, and well-located tumors are cured with surgery. In bitches with higher grades of tumors which have a predisposition for metastatic development, there is the opportunity to use additional therapy [100].

Radiotherapy is only used as an adjuvant co-treatment to surgery in dogs with inflammatory or metastatic carcinoma or patients with partially resected tumors [139]. In human medicine, radiation therapy plays an important role in many types of cancers, but in veterinary medicine, it has only been used for palliative treatment of different canine tumors (nasal lymphoma, carcinoma, or osteosarcoma) with promising results [140,141,142].

As mentioned above, ovarian hormones (progesterone and estrogens) belong to the risk factors for CMT development. Many mammary tumors in dogs demonstrate positivity for hormone receptors, especially well-differentiated carcinomas or benign epithelial tumors. Targeted therapy to inhibit hormone receptors may be a possible choice for CMT treatment [1]. This therapy includes modulators of estrogen receptors, luteinizing hormone-releasing hormone (LHRH) agonists, or progesterone antagonists [6]. Goserelin, which belongs to the group of LHRH agonists, caused a decrease in levels of estradiol and progesterone and a reduction of tumor size in dogs with CMTs [143]. In human medicine, tamoxifen, a non-steroidal blocker of estrogen receptors, is the most common drug used for treatment in women with estrogen receptor-positive breast tumors [144]. On the other hand, no anticancer activity has been reported in dogs after treatment with tamoxifen; only side effects such as vulvar discharge, nesting pyometra, and vulvar swelling were demonstrated [145]. Aglepristone, a progesterone antagonist, had an antiproliferative effect in progesterone receptor-positive CMTs [146]. However, further research on the use of hormonal therapy in dogs with CMTs is needed.

Dogs with CMTs can also be treated using different chemotherapeutic agents. Chemotherapy is used in patients with metastatic or inflammatory CMTs or as an adjuvant treatment to surgery [147]. The combination of several chemotherapeutic drugs, such as cyclophosphamide with 5-fluorouracil, or cyclophosphamide with mitoxantrone and vincristine, significantly increased survival time [147,148]. An overall response rate of 13% was found in patients treated with a combination of carboplatin and gemcitabine [149]. These combinations of cytotoxic drugs caused only mild side effects (gastrointestinal or hematological toxicity) [147,148,149]. Treatment of gemcitabine alone, docetaxel in combination with doxorubicin, or a combination of doxorubicin, cyclophosphamide, and 5-fluorouracil, showed no significant difference in overall survival time and time to metastasis [150,151,152].

One of the risk factors of CMTs, especially the malignant form, is an increased expression of COX-2 [85]. The use of COX-2 inhibitors as a potential therapy in different canine tumors has been described [84,153,154]. Anti-inflammatory drugs that inhibit COX-2 can be divided into two groups: non-selective and selective COX-2 inhibitors [155]. Nonsteroidal anti-inflammatory drugs (NSAIDs) belong to the non-selective group, because their mechanism of action involves the blockade of both COX isoenzymes. To avoid the adverse effects resulting from COX-1 inhibition, mainly gastrointestinal bleeding, selective COX-2 inhibitors, called, “coxibs”, were designed [156]. Experimentally, both non-selective NSAIDs and selective coxibs have been found to inhibit tumorigenesis by modulating apoptosis, suppressing cancer cell growth, inhibiting proliferation, and reducing the metastatic potential of cells [157,158]. The NSAIDs piroxicam and meloxicam have been able to induce cell cycle arrest, apoptosis, and migration suppression in vitro in CMT cells [159,160]. Moreover, piroxicam has been described as reducing tumor size in a xenograft model of CMT [161]. In a study by Souza et al. (2009), piroxicam increased survival time and improved clinical condition in dogs with inflammatory CMTs [152]. The effectiveness of coxibs in veterinary medicine has been tested in cell lines, mice xenograft models, and clinical trials. Celecoxib and mavacoxib showed cytotoxic activity, induction of apoptosis, and proliferation suppression in CMT cell lines [162,163]. A case-control prospective study demonstrated the ability of firocoxib to increase disease-free survival and overall survival in dogs with malignant CMTs [164]. Recently, Brandi and co-workers (2023) demonstrated the pro-apoptotic effect of firocoxib in CMT cell lines in vitro and in vivo; thus, it can be considered a potential neoadjuvant treatment for dogs with CMTs [165].

Metronomic therapy is an attractive approach to cancer treatment that involves the continuous administration of low doses of chemotherapy drugs at regular intervals, often without extended breaks. This anti-cancer treatment strategy has been explored and utilized in both human and veterinary medicine as a potential treatment option for various types of cancers, including breast cancer, mammary tumors, soft tissue sarcomas, and lymphoma. The key mechanism of metronomic therapy involves the continuous administration of low doses of chemotherapy drugs in combination with NSAIDs to target tumor angiogenesis, inhibit cancer cell proliferation, and stimulate the immune response against cancer. Cyclophosphamide (a chemotherapy drug) in combination with piroxicam (NSAID) and optionally with furosemide (a diuretic drug) is most commonly used in metronomic protocols in veterinary practice. Overall, metronomic therapy offers a convenient, cost-effective, and relatively low-risk option for the management of certain types of cancer in small animals. However, it is still necessary to keep in mind the potential benefits and risks, as well as close monitoring to ensure safe and effective treatment [166,167,168].

An important strategy of anticancer therapy is also anti-angiogenic treatment with monoclonal antibodies (mAbs), which is used in human oncology. The most widely used anti-angiogenic treatments are therapies against the VEGF family that can block ligands or receptors [169]. Bevacizumab, which was approved by the U.S. Food and Drug Administration (FDA) for human breast cancer treatment, was the first anti-angiogenic drug acting on VEGF [170]. Another mechanism of action of anti-angiogenic drugs is VEGF receptor blockade mediated by tyrosine kinase inhibitors, such as sorafenib, sunitinib, among others [171]. Sorafenib has been described as having an anticancer effect in vitro and suppresses neovascularization in a xenograft model of human breast cancer [172]. In the veterinary field, some mAbs have been tested in vitro for their anticancer potential in canine mammary cancer cells [166,167]. Prado and co-workers (2019) demonstrated the inhibitory effect of sorafenib on vasculogenic mimicry in canine mammary cancer cell lines in vitro [173]. Similarly, vandetanib showed promising results on a CMT model due to the inhibition of EGFR phosphorylation, resulting in reduced cell proliferation, migration, and VEGF production [174]. Moreover, another molecule with anticancer and anti-angiogenic activity on CMT cell lines is the carotenoid fucoxanthine, isolated from brown algae. Fucoxanthine inhibited cell migration and angiogenesis, induced apoptosis, and canceled cell death, suggesting that it could be a new potential agent for CMT treatment [175].

Lastly, in addition to drugs, oncolytic viruses can be also used as anticancer agents both in humans and dogs. Oncolytic virotherapy has been studied in recent years as a new strategy for cancer treatment. The possible mechanisms of action exerted by these viruses include immune-stimulatory effects and direct cytotoxicity to cancer cells [176]. Moreover, oncolytic viruses were able to target genes involved in the regulation of angiogenesis, namely EGF, angiopoietin-1, and angiopoietin-2 in breast cancer models, resulting in angiogenesis suppression [177]. In veterinary medicine, there are a few viruses that have been investigated as oncolytic agents, such as reovirus, vaccinia virus, adenovirus, measles virus, and canine distemper virus [178,179,180,181,182,183]. Gentschev et al. (2009) described that recombinant oncolytic vaccinia virus suppressed the proliferation of a canine mammary cancer cell line [179]. A few years later, Shoji and co-workers (2016) also demonstrated the antitumor activity of recombinant measles virus on a CMT model in vitro [181]. Similarly, the canine distemper virus-induced apoptosis and caused the growth inhibition of canine mammary adenocarcinoma cells [176]. Taken together, oncolytic viruses may be a possible choice for cancer therapy, but further in vivo and clinical studies are needed.

## 3. Personalized—Precision Medicine in Cancer Therapy

Cancers represent one of the leading causes of mortality worldwide. The number of cases increases every year, and the standard therapy is not effective in all patients. Traditionally, cancer diseases have been treated by general approaches, including surgery, radiotherapy, or chemotherapy. However, these treatment procedures often damage healthy noncancerous cells, and their efficacy varies widely across individual patients [184].

Tumors are characterized by different genetic materials and variability of the expressed proteins in one patient compared to another, resulting in disease heterogeneity. These inherent differences in cancer led to the development of personalized medicine [184]. The term “personalized or precision medicine” refers to an approach that involves the use of genetic information about each patient to achieve optimal therapeutic care for the patient. According to the National Research Council, the older term “personalized medicine” has been replaced by the term “precision medicine” with a similar meaning. However, there has been some misunderstanding with the word “personalized” because many people thought that unique treatments and preventions were being developed uniquely for each patient. Precision medicine is focused on finding approaches that will be effective for patients according to genetic, lifestyle, and environmental factors. Both terms are still used interchangeably [185]. The main goal of personalized medicine is to select the proper medication at the right dose and at the right time according to the patient’s specific needs [186]. Traditional personalized medicine was based on information about the patient’s family history, lifestyle, social circumstances, and environment. This is in contrast to modern personalized medicine based on targeted therapy, which studies the genetic, protein, and metabolic profile of patients in detail. The development of a new molecular assay that allows measurement of the levels of specific mutations, genes, and proteins is very important for diagnostics in this medical model [186].

Assessing the tumor microenvironment (TME) to enhance cancer treatment is a critical aspect of precision medicine. The TME is an intricate network that includes not only the actual tumor cells but also a variety of tumor-associated cells that are essential for both tumor progression and the efficacy of treatment. Tumor-associated cells and tumor cells interact by direct cell-to-cell contact, extracellular vesicles (microvesicles), and the production of soluble chemicals. These interactions can influence various aspects of tumor biology, including proliferation, apoptosis resistance, immune evasion, maintenance of stemness, invasion, and metastasis [187]. Hypoxia, or low O_2_ levels, is a characteristic feature of many cancers, and therefore O_2_ appears to be a relevant parameter of TME to investigate. Low oxygen levels are unfavorable for the survival of normal healthy cells. However, tumor cells can change gene expression and thus create a hypoxic response, which is their protective mechanism against cell death. Analysis of TME parameters, including biomarkers, O_2_ levels, and chemotherapeutic drug concentrations, throughout of a patient’s treatment schedule could influence the effectiveness of different therapies [188].

**Figure 5:**
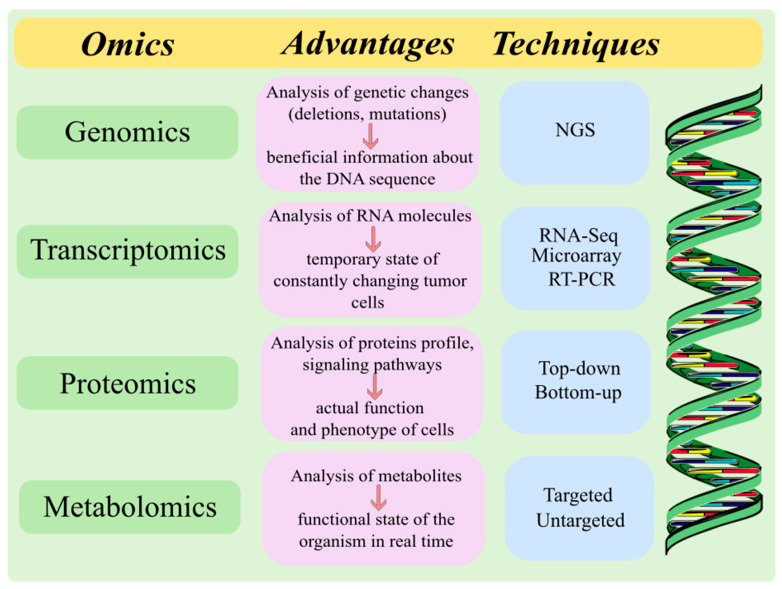
The major omics techniques for data acquisition used in personalized medicine. This original figure was created using the Inkscape v1.1.2 software.

The first and most important step in the development of personalized therapy is acquiring data about the patient (Figure 5). The individual disease state of the patient is characterized by several omics techniques (genomics, proteomics, metabolomics, and transcriptomics) [186], and each of them has some advantages and limitations. Analysis of the interactions among these molecular approaches helps to understand personalized oncology [189]. Cancer genomics aims to determine new genetic changes for a specific type of tumor using DNA sequencing and analysis of the patient’s cancer cells. Genomic data are most often collected using next-generation sequencing (NGS) technologies [184]. Data about genomic variations (deletions, mutations, copy number changes, single nucleotide polymorphisms) in different cancers are leading to the development of more effective strategies to prevent, diagnose, and treat cancers [186]. Analysis of the tumor genome provides beneficial information about the DNA sequence but does not adequately describe the actual cells phenotype [190]. Therefore, other omics techniques are needed to find specific treatment for the individual cancer patient [189].

Transcriptomics technologies represent the field of molecular genetics which also contributes to personalized medicine. The transcriptome is the set of all RNA molecules (protein-coding mRNAs and non-coding RNAs) that were transcribed from the genome in the cells. Every RNA molecule has its functions in the cells and can react actively to environmental stimuli [191]. The transcriptome is considered the crosstalk between genetic aspects and the cellular phenotype. Furthermore, the genome is relatively stable compared to the transcriptome, which reflects the temporary state of the cell. Therefore, transcriptomics can be useful for molecular analysis of constantly changing tumor cells and contribute to the rapid development of precision and personalized oncology [189]. RNA-Sequencing (RNA-Seq), microarray analysis, and Large-Scale Real-Time Reverse Transcription PCR (RT-PCR) are major methods for collecting transcriptomics data [192]. Microarray technology is the most frequently used approach for the analysis of the transcriptome. However, both microarray and RT-PCR require prior knowledge about gene sequences. Distinct from the above-mentioned methods, sequencing-based techniques such as NGS is referred to nowadays as the “gold standard” of transcriptome profiling. RNA-Seq allows the molecular mechanisms involved in oncogenesis to be analyzed and the investigation of thousands of genes, thereby contributing to the development of personalized medicine [189].

Although the capability of biological and molecular functions is coded by genes, proteins are responsible for performing actual functions and more closely determine the phenotype of cells. Proteomic measurements include information about protein synthesis, concentrations, structure, degradation, interactions between proteins, and cellular localization [193]. Two major methods have been investigated for collecting proteomic data: top-down and bottom-up proteomics. The bottom-up approach is used to analyze a mixture of proteins and their composition using mass spectrometry (MS). The newer approach, the top-down strategy, uses MS for analyzing whole proteins and identifies post-translational modifications, which is important for personalized medicine therapy [184]. The Human Proteome Project compares the peptides and proteins profile in a healthy person to those of a cancer patient. The assays for cancer detection and the major signaling pathways in tumor development are based on protein level. Moreover, most of the drugs used in the treatment are directed towards proteins. For these reasons, proteomic information is very important for personalized medicine [186].

Another branch of personalized medicine is metabolomics, which studies small molecules produced during metabolic reactions. Metabolites reflect genetic as well as environmental influences, and a complete analysis of the metabolome describes the functional state of the organism in real time. The clinical advantage of metabolomics is that metabolites can be detected noninvasively due to their diffusion throughout the body and easy availability in biofluids like urine or blood [194]. Over the past decade, MS analysis has been used to identify metabolites due to its sensitivity to low concentrations and high resolution. The collection of metabolomics data uses two major approaches, untargeted and targeted. As in proteomics, an untargeted strategy is often used in studies to analyze metabolites that are linked to the cancer phenotype. On the other hand, targeted metabolomics determines metabolites present in patient samples. Therefore, metabolomics is a promising strategy in personalized medicine therapy [184].

Analysis of the obtained data is important for the development of oncology drugs and the forthcoming treatment of cancer disease. Advancements in omics techniques help discover new products of personalized medicine, including mAb, cancer vaccines, and chimeric antigen receptor (CAR) T-cells. Methods for detecting circulating tumor cells (CTCs) and DNA have promising potential for early diagnosis, risk monitoring, and identification of effective treatment for the individual patient. Several therapies focused on the immune system, whose aim is to improve specific immune responses, can lead to effective, powerful, and personalized cancer treatment [184].

The mAbs are high molecular weight proteins that can bind to a specific target molecule expressed in a particular tumor. They seem to be promising in cancer treatment, due to their high specificity and low toxicity [195]. For instance, HER-2 positive breast cancers with positivity to HER-2 have better clinical results than HER-2 targeted mAb. The choice of mAb is influenced by the cancer type and subtype and information about overall efficacy and adverse effects from previous clinical trials [184]. It is generally known that tumor cells tend to evade the T-cell immune response. They can overexpress several ligands, which activate T-cell inhibitory receptors. T-cell surface receptors or “immune checkpoints” are responsible for suppressing the immune response of cytotoxic T-cells. The development of antibodies with the ability to block “immune checkpoints” has a promising potential in cancer treatment [196].

A novel strategy of cancer immunotherapy is the use of cancer vaccines, which can increase the intensity of tumor-specific T-cell response based on active immunization. Tumor neoantigens discovered using NGS and other omics techniques have a central role in tumor vaccination. Typical features of these neoantigens are their high tumor specificity because they arise from somatic mutation of the tumor. Identification of divergent tumor neoepitopes among individual patients is important for the development of personalized cancer vaccines [197].

Another personalized approach to cancer treatment is CAR T-cell therapy, which directly primes the cells of an individual patient to better fight their cancer. Autologous CAR T-cells are prepared to express a CAR, which specifically targets and damages malignant cells or releases soluble factors that remodel the tumor microenvironment [198]. Data collection about different tumor genotypes and phenotypes contributes to the optimization and individualized design of CAR T-cell-based therapies [199]. CAR T-cell therapy has shown promising results in the treatment of hematologic malignancies, such as B-cell lymphoma or acute lymphoblastic leukemia. On the other hand, CAR T-cell therapies have had limited success in different solid tumors [200].

### Personalized Medicine in Veterinary Oncology

Personalized medicine is one of the newest approaches used in human oncology, but it has not yet been sufficiently explored in the veterinary field. Studies focused on the proteomic profile and mRNA expression in cancers (mainly canine) have already provided a few clinically relevant biomarkers and mRNA patterns, which can be considered as a starting point for the use of personalized medicine in veterinary oncology [201].

The closest example of personalized therapy use in dogs is the tyrosine kinase inhibitors (toceranib, masitinib, and imatinib) that were approved by the FDA for the treatment of canine mast cell tumors [202]. Toceranib exerts anticancer activity through inhibition of the c-Kit receptor against a broad spectrum of canine cancers, particularly in dogs with pulmonary metastases [203]. Toceranib also blocks VEGFR, thereby having an anti-angiogenic effect. Moreover, it can increase immune surveillance by producing a significant decrease in regulatory T-cells [204]. CMT cell lines have shown a reduction in cell proliferation after toceranib treatment [205]. Another selective inhibitor of the c-Kit receptor is masitinib, which is used in the treatment of canine mast cell tumors [206]; however, there are no studies on CMT therapies [155]. Recent studies investigated potential “actionable” genotypes in different canine tumors including melanoma [207], hemangiosarcoma [208], osteosarcoma [209], and others, but clinical trials are needed to confirm their effectiveness [210].

Additionally, some mRNA patterns with prognostic potential have also been introduced. Microarray technology determined that metastatic potential and malignancy levels of CMTs and lymphomas are reflected in their transcriptome [211,212]. The study of Frantz et al. (2013) identified an association between mRNA pattern and the grade, immunophenotype, and therapeutic response of canine lymphomas [213]. In addition, several studies have focused on biomarkers identified in canine neoplasms using a proteomics approach. Potentially relevant biomarkers for lymphomas or mammary tumors have been found in canine serum [214,215]. However, the results of these experiments have not yet been translated into clinical assays. Methods used for the detection of CTCs in CMT also contributed to the improvement of personalized therapy in the veterinary field. The presence of CTCs significantly correlated with the metastatic potential of CMTs [216]. A broader analysis of biomarkers and mRNA patterns can be useful for the development of personalized therapy in veterinary oncology.

A novel and interesting approach of personalized oncology in the veterinary field is immunotherapy (Table 3). Studies revealed that canine mammary cancer is considered the best-known homologous animal model to human breast cancer due to their similarities in epidemiology, pathology, and immunohistochemical characterization [217,218]. The antigens HER-2 and MUC-1 are the most well studied antigens in human breast cancer. Razazan et al. (2017) demonstrated that nanoliposomes conjugated with HER-2-derived peptides acted as effective vaccines against mammary cancer in mice models. These vaccines suppressed tumor growth and induced high levels of CD8^+^ cells, interferon-γ (IFN-γ), and CTL response [219]. Similar results were also shown in the study of Farzad et al. (2019), where another HER-2-derived peptide conjugated with liposomes increased CTL reactions, resulting in a lower tumor size and higher survival time in the vaccinated group of a murine mammary cancer model [220]. Moreover, targeting HER-2 with trastuzumab was described as causing the growth suppression of canine tumor cells, indicating that targeting HER-2 seems to be an important approach in personalized immunotherapy development [221]. Another type of DNA vaccine that has been investigated is the MUC-1 vaccine, which was able to decrease tumor growth in mice bearing mammary cancer [222]. A MUC-1/HER-2 chimeric protein induced cellular and humoral immune response in a murine breast cancer model. Furthermore, this vaccine reduced lung metastasis and increased cancer cell death [223]. It has also been reported that mAb against MUC-1 can be an important diagnostic and therapeutic agent in mammary cancer [47,224].

As mentioned above, CAR T-cells represent an interesting research area in cancer therapy. Since MUC-1 is aberrantly glycosylated and overexpressed in more than 90% of mammary tumor cases, it is considered a favorable target for engineering CAR T cells. Zhou and co-workers (2019) showed a significant anticancer and cytotoxic potential of anti-MUC-1 specific CAR T-cells in triple-negative breast cancer in vitro and in vivo [225]. 

Immunotherapeutic approaches including DNA vaccines, mAbs, and CAR T-cells have promising potential to become additional therapeutic options for CMT and thus contribute to the development of personalized medicine.

## 4. Conclusions

Nowadays, animals with spontaneous tumors are mostly used as a suitable model for research on human cancer disease [226]. CMT represents one of the major health problems in dogs, and due to its characteristics, including size similarities, rapid growth, and identical clinical stages to human breast cancer, it is considered an interesting choice for comparison studies [218,227]. The important risk factor in the etiology of CMT is ovarian steroids; therefore, ovariohysterectomy of female dogs at an early age is the most successful means for preventing mammary tumors. Only some dogs undergo preventive ovariohysterectomy, however, which leads to CMTs often being detected at a progressive stage and thus having a poor prognosis. Moreover, they are still insufficiently studied and therapeutic options are lacking. For these reasons, early diagnosis of CMTs, new therapeutic strategies, and the development of personalized medicine could be beneficial. The continuous identification of new biomarkers and the use of personalized treatment seem to be highly favorable mainly in cancer disease. The extensive variability among patients with tumors emphasizes the need to target each individual in a personalized manner. Unfortunately, data on personalized approaches in veterinary oncology are still lacking. However, initial recent studies focused on mRNA expression or proteome changes in canine mammary models suggest that personalized therapy is a new important challenge for future studies and may soon become a reality in veterinary oncology.

## Figures and Tables

**Table 1:** Classification of canine mammary tumors.

** 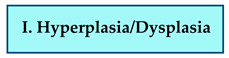 **	Duct ectasia
	Lobular hyperplasia (adenosis)
	Epitheliosis
	Papillomatosis
** 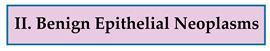 **	Simple benign tumors
	Non-simple benign tumors
	Ductal-associated benign tumors
** 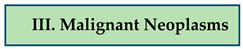 **	Simple carcinomas
	Non-simple carcinoma
	Ductal-associated carcinoma
** 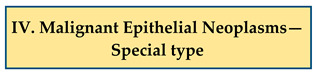 **	Squamous cell carcinoma
	Adenosquamous carcinoma
	Mucinous carcinoma
	Lipid-rich (secretory) carcinoma
	Spindle cell carcinoma
	Malignant myoepithelioma
** 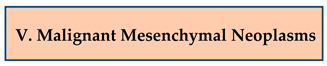 **	Osteosarcoma
	Chondrosarcoma
	Fibrosarcoma
	Hemangiosarcoma
	Other sarcomas
** 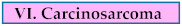 **	
** 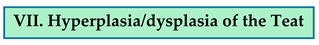 **	Melanosis of the skin of the teat
	Hyperplasia of the teat
** 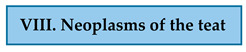 **	Benign ductal-associated neoplasms
	Malignant ductal-associated neoplasms
	Carcinoma with epidermal infiltration (Paget-like disease)

**Table 2:** Histological grading system of canine mammary cancer.

Grade of Malignancy	Tumor Differentiation	Total Score
**I.**	well-differentiated	3–5
**II.**	moderately differentiated	6–7
**III.**	poorly differentiated	8–9

**Table 3:** Immunotherapy for personalized veterinary medicine.

** 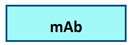 **	Toceranib
	Masitinib
	Imatinib
** 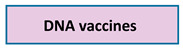 **	Nanoliposomes conjugated with HER-2-derived peptide
	MUC-1 vaccine
	Chimeric MUC-1/HER-2 vaccine
** 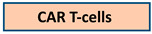 **	Anti-MUC-1 specific CAR T-cells

## Data Availability

No new data were created or analyzed in this study. Data sharing is not applicable to this article.
